# Horizontal Guided Bone Regeneration of the Posterior Mandible to Allow Implant Placement: 1‐Year Prospective Study Results

**DOI:** 10.1111/clr.14363

**Published:** 2024-10-01

**Authors:** Jonas Lorenz, Shahram Ghanaati, Zoran Aleksic, Iva Milinkovic, Zoran Lazic, Marko Magić, Bastian Wessing, Ramona Schleich Grotenclos, Mauro Merli, Giorgia Mariotti, Eriberto Bressan, Luca De Stavola, Robert Sader

**Affiliations:** ^1^ FORM—Frankfurt Orofacial Regenerative Medicine, Clinic for Oral, Cranio‐Maxillofacial and Facial Plastic Surgery Medical Center of the Goethe University Frankfurt Frankfurt am Main Germany; ^2^ Department of Periodontology and Oral Medicine, School of Dental Medicine University of Belgrade Belgrade Serbia; ^3^ Department of Implantology Medical Military Academy Belgrade Serbia; ^4^ School of Dental Medicine University of Belgrade Belgrade Serbia; ^5^ Praxisklinik der Zahnheilkunde am Luisenhospital Aachen Germany; ^6^ Clinica Merli Rimini Italy; ^7^ Department of Neurosciences, School of Dentistry University of Padova Padova Italy

**Keywords:** alveolar ridge augmentation, bone regeneration, bone substitutes, dental implant, torque, xenograft

## Abstract

**Objective:**

Assess whether horizontal ridge augmentation with guided bone regeneration (GBR) using deproteinized bovine bone mineral (DBBM), autologous bone, and a resorbable collagen membrane supports successful implant placement.

**Materials and Methods:**

This open, prospective, single‐cohort, multicenter clinical study included patients with ridge defects that required GBR prior to implant insertion. The primary endpoint was radiologically assessed bone gain after 8 months post‐GBR, measured at the center of planned implant sites. Secondary endpoints included implant survival and success, marginal bone levels (MBLs), MBL changes, and soft tissue health.

**Results:**

Of 45 patients evaluated 8 months post‐GBR, nine experienced dehiscence in the first 3 weeks of the healing period. GBR led to radiologically determined mean bone width gain of 4.0 ± 1.5 mm and 4.8 ± 1.7 mm, measured 1 and 3 mm from the top of the crest, respectively, allowing successful implant placement in 44 patients (97.8%). The cumulative implant survival and success rates were 98.9% and 95.5%, respectively. MBLs were stable: −1.18 ± 0.64 mm at definitive prosthesis placement (DPP) and − 1.07 ± 0.74 mm at 1 year. Soft tissue health and esthetics (plaque and bleeding indices, papilla, keratinized mucosa, and pink esthetic score) improved from DPP to 1 year. Patients were highly satisfied with implant function and esthetics, and their oral health‐related quality of life improved.

**Conclusions:**

GBR using DBBM and a collagen membrane offered a safe and effective treatment option for horizontal ridge augmentation sufficient to support implant‐based tooth rehabilitation.

**Trial Registration:**

Registered at ClinicalTrials.gov NCT03028922 (registrations sites, as above listed affiliations, first posted January 23, 2017)

## 1 Introduction

After tooth loss, the bone naturally resorbs, creating alveolar ridge defects (Araujo, Wennstrom, and Lindhe 2006; Cadenas‐Vacas et al. 2021; Schropp et al. 2003). When bone atrophy occurs, a grafting procedure might be necessary to improve the quality and quantity of bone before implant placement to ensure sufficient buccal bone thickness, which has been postulated to determine the extent of bone remodeling (Buser, Sennerby, and De Bruyn 2017; Monje et al. 2023).

Various bone‐grafting options are available, and all current methods, including autografts, allografts, xenografts, and synthetic bone grafts, are associated with both advantages and disadvantages. Xenogenic bone substitutes are often preferred by clinicians because they tend to undergo slower resorption than synthetic materials, allowing for volume maintenance; present with similar characteristics as human bone; have good osteoconductivity; and promote uneventful healing (Godoy et al. 2021; Lee et al. 2017; Thieu et al. 2021). Bone augmentation utilizing xenograft substitutes is considered a favored treatment option to support successful implant placement and osseointegration, where guided bone regeneration (GBR) represents a potential solution for patients with ridge defects (Park et al. 2005, 2007; Shin et al. 2014). The GBR approach utilizes a deproteinized bovine bone mineral (DBBM) that is tightly packed into the defect area and protected by a collagen membrane fixed with titanium pins. The membrane serves as a barrier, facilitates graft containment, and provides mechanical stability to the graft (Cadenas‐Vacas et al. 2021; De Bruyckere, Cabeza, et al. 2020; Reddy et al. 2019; Redemagni, Mascetti, and Garlini 2019; Wessing, Emmerich, and Bozkurt 2016; Wessing et al. 2017), which is often necessary with larger ridge defects (Sanz et al. 2019) or defects that require more complex remodeling than the simple filling of a fresh extraction socket. Xenografts using DBBM and a resorbable, non‐crosslinked membrane consisting of collagen and elastin fibers provide a high degree of mechanical strength when immobilized using pins or sutures, yielding uneventful wound healing that allows for nutrient flow and proper vascularization (Bozkurt et al. 2014; Cadenas‐Vacas et al. 2021; De Bruyckere, Cabeza, et al. 2020; De Bruyckere, Cosyn, et al. 2020; Park et al. 2005, 2007; Reddy et al. 2019; Redemagni, Mascetti, and Garlini 2019; Sanz et al. 2019; Shin et al. 2014; Wessing, Emmerich, and Bozkurt 2016). Multiple other approaches, such as ridge splitting or the use of L‐blocks, can be employed together to enhance graft stability, and the optimal combination depends on ridge defect conditions. The main alternative strategy combines GBR and simultaneous implant placement, using pins to fix the membrane in place and a supplementary L‐shaped bone block (Mir‐Mari et al. 2016). However, bone blocks tend to vascularize at a slower rate than particulate grafts, may retain cellular components (Lorenz et al. 2017; McAllister and Haghighat 2007), and may be difficult to fit, making them prone to fractures.

Despite a large body of research describing bone gain after GBR, a recent review by Smeets et al. (2022) reported that few studies use a systematic approach to describe the outcomes of various horizontal augmentation approaches in the lower jaw; they may not include an adequate number of specified mandibular patients or conduct follow‐up beyond 6 months. The mandible contains poorly vascularized cortical bone, which may make some techniques, such as bone splitting and spreading, particularly challenging (Bassetti, Bassetti, and Bosshardt 2016).

This study examined the effectiveness of DBBM and a resorbable collagen membrane for treating critical ridge defects while closely monitoring patient satisfaction scores. Most of the treated defects corresponded to more than one missing tooth, requiring a high degree of structural stabilization to enable restoration of up to three teeth. The primary endpoint was radiologically assessed bone gain after 8 months post‐GBR, measured at the center of the planned implant sites, with secondary outcomes including soft‐tissue measures. In addition, a sub‐analysis of patients who experienced dehiscence was performed, and the patient experience was recorded.

## 2 Materials and Methods

### 2.1 Ethical Considerations

All study procedures were conducted in accordance with the principles established in the Declaration of Helsinki and were approved by the appropriate review board at each participating center: Ethical Committee at the Department of Medicine, Goethe University Frankfurt (474/16); Ethical Committee at Medical Military Academy, Belgrade (August 29, 2016); Ethical Committee for Clinical Experimentation of the Province of Padova (3851/AO/16); Ethical Committee IRST IRCCS AVR Emilia‐Romagna Region (5827/2016 I.5/130); Ethical Committee of Medical Association of Nordrhein (2016233); and Ethical Committee of the School of Dental Medicine University of Belgrade (36/34). All patients provided written informed consent prior to the performance of any study procedures, and only data collected after the patients had signed informed consent were considered during the study analysis.

### 2.2 Study Design and Participants

An open, prospective, single‐cohort, multicenter clinical study was conducted to evaluate bone gain at sites requiring GBR prior to implant placement in the premolar and molar regions of the mandible after horizontal augmentation using a DBBM indicated for GBR (creos xenogain, Nobel Biocare AB, Göteborg, Sweden), together with a resorbable collagen membrane made of non‐crosslinked porcine‐derived collagen and elastin fibers that has a low surface expansion when hydrated (creos xenoprotect, Nobel Biocare AB).

Six centers (private practices and hospital‐based clinics in Germany, Italy, and Serbia) participated in this study. The first patient visit occurred on October 18, 2016, and the patient recruitment phase was finalized on May 19, 2017. The last follow‐up assessment was completed on March 12, 2020, when the last patient attended their last visit. Each investigator received training on the study protocol and requirements, performed at each participating center, prior to the study start.

Patients underwent clinical assessments of the mandibular alveolar ridge dimensions to evaluate bone deficiency using low‐dose computed tomography (CT) or cone‐beam CT (CBCT) at the planned implant level. Horizontal measurements were performed at 1.0 and 3.0 mm from the top of the crest, and vertical measurements were defined as the distance from the anatomical landmark (alveolar nerve or lingual dehiscence) to the top of the crest. If the bone volume at the center of the planned implant site (the reference point) was insufficient for implant placement, the subject was designated as requiring a GBR procedure and was considered a candidate for the study. In addition, UNC 15‐based measurements were performed at the patient level, over the center of the augmentation site. Radiological results were chosen because they could be well‐documented and examined by an experienced, independent expert, enabling the elimination of inter‐center and inter‐clinician variations. The CBCT measurement covered FDI positions, with 1–3 positions per subject. An anatomical landmark (alveolar nerve or lingual dehiscence) was used to align the pre‐ and post‐augmentation CBCTs.

In addition to the initial screening, inclusion criteria were age of 18–80 years; provided written informed consent; in need of horizontal ridge augmentation in the posterior region of the mandible prior to implant placement; medium (4–6 mm) or large (≥ 7 mm) horizontal defect or combination horizontal/vertical defect with ≤ 2 mm loss in the vertical dimension; good physical health (i.e., controlled/treated or absence of serious illnesses); good oral hygiene; willing and able to comply with all study‐related procedures; full‐mouth bleeding score ≤ 25%; full‐mouth plaque index ≤ 25%; and suitable for 2‐stage surgical procedure. Exclusion criteria included prior bone augmentation in the planned treatment area; health conditions that do not permit the performance of any stage of surgical or restorative procedures; disorders in the planned implant area (e.g., tumors or chronic bone disease); use of interfering medications (e.g., steroid or bisphosphonate therapy); history of drug or alcohol abuse; heavy smoking (> 10 cigarettes per day); possibility that treatment may negatively affect overall health (e.g., psychiatric problems); uncontrolled diabetes; poor compliance; active periodontal disease (pocket probing depth of at least 5 mm, accompanied by bleeding‐on‐probing and signs of inflammation) involving residual dentition; or mucosal disease in the treated area. All defects were located at healed sites defined as at least 6 months post‐extraction and were extrabony.

If a subject required a bilateral GBR procedure in the premolar or molar regions of the mandible and both ridge dimensions fulfilled the inclusion criteria, only the first procedure was included in the analysis. Although the amount of residual keratinized mucosa (KM) was not an inclusion/exclusion criterion, KM < 2 mm was a deciding factor for the 2‐stage protocol.

### 2.3 Initial Augmentation Protocol

Bone quality was assessed during the GBR procedure and scored as follows: (1) almost the entire jaw is comprised of homogenous compact bone; (2) a thick layer of compact bone surrounds a core of dense trabecular bone; (3) a thin layer of cortical bone surrounds a core of dense trabecular bone of favorable strength; or (4) a thin layer of cortical bone surrounds a core of low‐density trabecular bone. Bone quantity was also assessed and scored, as described previously (Lekholm and Zarb 1985): (A) most of the alveolar ridge is present; (B) moderate residual ridge resorption has occurred; (C) advanced residual ridge resorption has occurred, and only basal bone remains; (D) some resorption of the basal bone has started; or (E) extreme resorption of the basal bone has occurred. Autogenous bone was harvested by scraping bone chips from the retromolar area using a single‐use scraper; if necessary, bone chips were further particulated prior to being mixed with DBBM granules at a 1:1 ratio. A full crestal incision in the keratinized gingiva on the alveolar crest was created, together with one or two vertical releasing incisions. All vertical incisions were placed at least one tooth away from the surgical site. Using periosteal elevators, a full‐thickness flap was created to expose the recipient bone bed, and a periodontal probe with millimeter markings was used to measure the available crest at baseline. Multiple decortication holes were created on the ridge using a small round bur to improve blood supply in the area. After placement of the mixed graft substrate, a porcine, resorbable, non‐crosslinked membrane adjusted to the defect size was used to cover and immobilize the mixed graft substrate and fixed with titanium pins. The flap was then sutured with a primary closure, using first horizontal mattress sutures and then single interrupted sutures. No soft tissue grafting was performed. Because this study had a multicenter design, the antibiotic and postsurgical regime followed standard care procedures established at each treating clinic. Due to the wide spectrum of defect sizes, for uniformity, all regenerated areas were subjected to the same 8‐month healing period to allow sufficient time for those that were more extensive.

### 2.4 Histology

Immediately before implant insertion, a trephine drill (Meisinger, 2.0/3.0 mm inner/outer diameter) was used to obtain a histological sample that was placed in freshly prepared 4% buffered formalin (pH 7–7.5) for 48 h at room temperature. The fixative volume‐to‐tissue ratio was at least 10:1. The biopsy was dehydrated in ethanol and embedded in Kulzer Technovit 7200 VLC (Kulzer GmbH, Hanau, Germany) according to standard procedures and sectioned at a thickness of 60 μm. Sections were prepared according to the Donath technique (Donath and Breuner 1982), stained with Sanderson's Rapid Bone Stain (Dorn & Hart, Loxley, AL, USA), and counterstained with acid fuchsin (Sigma, St. Louis, MO, USA) to visualize mineralized and non‐mineralized tissues.

### 2.5 Implant Insertion and Restorative Procedures

NobelParallel Conical Connection (Nobel Biocare AB) implants were used according to the manufacturer's instructions, with the following considerations: minimal implant length of 8.5 mm; implant diameters of 3.75, 4.3, or 5.0 mm; and minimum ridge width (RW) to allow 1.5 mm from the buccal and lingual sides to the implant. Implants were placed subcrestally or equicrestally using an insertion torque not to exceed 45 Ncm. Typically, at least 2 mm of KM was required (Esfahanizadeh et al. 2016; Moraschini et al. 2017) to qualify for 1‐stage surgery (transmucosal healing) using a healing or multi‐unit abutment (MUA); otherwise, a 2‐stage surgery with submerged healing was performed. No soft tissue grafting was performed simultaneously with implant placement. The approach to healing and the possibility of provisional restoration were determined at the clinician's discretion and according to routine clinical practice. At the time of definitive prosthesis placement (DPP), various retention modes and types of esthetic abutments were used, including the NobelProcera esthetic abutment or a temporary abutment with ceramic veneering.

### 2.6 Outcome Measures

The outcome measures collected in this study follow the recommendations by Tonetti et al. (2023). Specifically, three core areas were assessed: pathophysiology (esthetics, surgical morbidity and complications, peri‐implant tissue health), lifespan (survival and complications), and life impact (quality of life and satisfaction with treatment). The outcomes associated with ridge size before and after augmentation were evaluated at the planned implant site level. If the patient required a bilateral augmentation, only the first procedure was included in the study. Outcomes associated with implant placement and subsequent follow‐up were also analyzed at the implant level.

### 2.7 Healing Phase

Soft tissue healing was evaluated at 1, 3, 12, and 24 weeks and 8 months after GBR. Potential complications during the healing period following bone augmentation include swelling at the surgical site, flap sloughing, bleeding, local inflammation, bone loss, infection, or pain. The treating clinicians were asked to describe healing as eventful or uneventful; sites were assessed for signs of infection (pus), and swelling was rated on a 0–3 scale (0, none; 1, minor; 2, moderate; 3, severe). Patient‐reported pain perceptions were assessed 1, 3, 12, and 24 weeks after GBR and at the time of implant insertion.

### 2.8 Primary Endpoint

Bone width gain was assessed after an 8‐month healing period using a CBCT to determine whether the ridge augmentation using GBR yielded sufficient volume to accommodate the planned implant.

### 2.9 Secondary Outcome Measures

#### 2.9.1 Implant Stability, Survival, and Success

The mobility of each implant was assessed at insertion by individual tapping or rocking with a hand instrument (Mombelli et al. 1987). Implants were classified as stable or unstable. Implant survival was registered from implant insertion to the 1‐year follow‐up (FUP) visit; surviving implants were defined as those that remained in the jaw or could be restored if damaged. Implant success was defined according to the criteria proposed by van Steenberghe (1997) and was registered from implant loading to the 1‐year FUP visit. Implants were considered unsuccessful if they caused allergic, toxic, or gross infectious reactions, either locally or systemically; failed to offer anchorage to a functional prosthesis; showed any signs of fracture or bending; showed signs of peri‐implant radiolucency on an intra‐oral radiograph using a paralleling technique strictly perpendicular to the implant‐bone interface; or showed mobility when individually tested by tapping or rocking with a hand instrument.

#### 2.9.2 Marginal Bone Levels and Marginal Bone Level Changes

Radiographic examination was performed using a standardized long‐cone parallel technique with a custom‐made bite block made after implant placement. Radiographic images could be collected digitally or conventionally, and double films were collected for all conventional radiographs. To prevent inter‐rater variability, all bone height measurements were analyzed by an independent experienced radiologist. Radiographic evaluations were conducted to assess the marginal bone level (MBL) at DPP, 6‐month FUP, and 1‐year FUP. The reference point used for the radiographic assessment was the implant platform. Images had to be perpendicular to the implant with a clear thread profile and at least 2 mm of surrounding bone visible. The bone level was measured as the distance between the most apical bone level to the implant–abutment junction using Adobe Illustrator. The distance was calibrated to the implant diameter. Bone levels were recorded mesially and distally, and the mean was calculated for each implant.

#### 2.9.3 Soft Tissue Health and Esthetics

Soft tissue health and esthetics were evaluated using the bleeding and plaque indices, papilla, KM assessment, and pink esthetic score (PES). The bleeding tendency was assessed by a modified Sulcus Bleeding Index (mBI) according to Mombelli et al. (1987) and scored as follows: 0, no bleeding occurred when a periodontal probe was passed along the gingival margin adjacent to the implant; 1, isolated bleeding spots were visible; 2, blood formed a confluent red line on the margin; or 3, heavy or profuse bleeding occurred. Plaque accumulation was assessed using the modified Plaque Index (mPI) according to Mombelli et al. (1987), as follows: 0, no detectible plaque; 1, plaque recognized when running a probe across the marginal implant surface; 2, plaque apparent to the naked eye; or 3, an abundance of soft matter. Both indices were rated at 6‐month and 1‐year FUPs.

Papillae were evaluated at DPP, 6‐month FUP, and 1‐year FUP using the Papilla Index according to Jemt (1997), as follows: 0, no papilla was present, with no indication of a soft tissue contour adjacent to the implant restoration; 1, less than half of the papilla height was present, and a convex curvature of the soft tissue contour adjacent to the implant crown and the adjacent tooth was observed; 2, half or more of the papilla height was present but did not extend to the contact point between the teeth, and the papilla was not harmonious with the adjacent papilla between permanent teeth; 3, the papillae filled the entire proximal space, were harmonious with the adjacent papillae, and presented an optimal soft tissue contour; or 4, the papillae were hyperplastic and covered too much of the implant restoration or adjacent tooth, with an irregular soft tissue contour. The investigators scored the Papilla Index only at either the mesial or distal sides of each implant position at each time point. The status of KM surrounding the implants was recorded as follows: 0, no KM around the implant; 1, the mucosa surrounding the implant was partially keratinized; or 2, the entire mucosa surrounding the implant was keratinized. KM height (in mm) was measured at the buccal side from the mucosal margin to the mucogingival junction from the implant. Both KM status and KM height were evaluated at DPP, 6‐month FUP, and 1‐year FUP.

Photographs were obtained to assess PES at DPP, 6‐month FUP, and 1‐year FUP. Despite the posterior position of these sites, PES was monitored in this study because it provides a validated assessment of peri‐implant soft tissue health. PES was determined by an experienced, independent examiner based on seven variables: mesial papilla, distal papilla, soft tissue level, soft tissue contour, alveolar process deficiency, soft tissue color, and soft tissue texture (Furhauser et al. 2005). Each variable was assigned a score between 0 and 2, with 2 being the best and 0 being the worst. The mesial and distal papillae were evaluated for completeness, incompleteness, or absence. All other variables were assessed by comparison with a neighboring reference tooth. The highest possible score of 14 reflects a perfect match between the peri‐implant soft tissue and that of the reference tooth, according to Furhauser et al. (2005). Adverse events were recorded as they occurred throughout the study duration.

### 2.10 Patient‐Reported Outcome Measures (PROMs)

Patients were asked to complete the Oral Health Impact Profile‐14 (OHIP‐14, translated into the local language) to document their perceptions of quality of life. The survey was assessed throughout the study, including pre‐treatment; 1, 3, 12, and 24 weeks after GBR; at implant insertion; and at DPP, 6‐month FUP, and 1‐year FUP. The OHIP‐14 score was calculated by totaling the scores for each question (Q1–14) for each patient and averaging them at each time point. Each question can be scored from 0 to 4 points, and the total OHIP‐14 score ranges from 0 to 56, with lower scores indicating better quality of life. Subjective measures of esthetic and functional satisfaction (Belser et al. 2004) were assessed at DPP, 6‐month FUP, and 1‐year FUP using a Visual Analog Scale (VAS) ranging from 1 (not satisfied) to 10 (fully satisfied). Each patient's subjective pain was assessed using a VAS, ranging from 0 (no pain) to 10 (very intense pain).

### 2.11 Statistical Analysis

The sample size was calculated based on the findings of a retrospective study by Urban, Nagursky, and Lozada (2011). A sample size of 40 was determined to provide 80% power to detect a difference in bone gain of 1.10 mm after 8 months at a significance level of 0.05. Accounting for a 5% dropout rate, 42 patients were targeted for enrollment. The calculation was based on a two‐sided Fisher permutation test. Horizontal bone gain was assessed using a one‐sample *t*‐test. All variables are described using frequencies [*n* (%)] or parametric descriptive statistics [mean ± standard deviation (SD)]. Cumulative survival and success rates are derived from Kaplan–Meier survival analyses, using the implant insertion date as the baseline. The relationship between the initial ridge width vs. horizontal bone gain and vertical height maintenance was tested by linear regression. The influence of crestal position at placement and 1‐ vs. 2‐stage surgery on marginal bone remodeling was assessed using the Kruskal–Wallis test. The relationship between KM status and bleeding index and oral health‐related quality of life was assessed with independent sample two‐sided *t*‐tests. Additional descriptive subanalysis was conducted to examine the potential relationship between wound dehiscence and outcome measures as well as defect size, implant size, or implant number per augmentation site. Calculations were performed using IBM SPSS Statistics software (Version 24 or greater, SPSS Inc., Chicago, IL, USA).

### 2.12 STROBE Compliance Statement

This manuscript is compliant with the Strengthening the Reporting of Observational Studies (STROBE) guidelines (von Elm et al. 2008).

## 3 Results

### 3.1 Study Flow and Participant Characteristics

**FIGURE 1:**
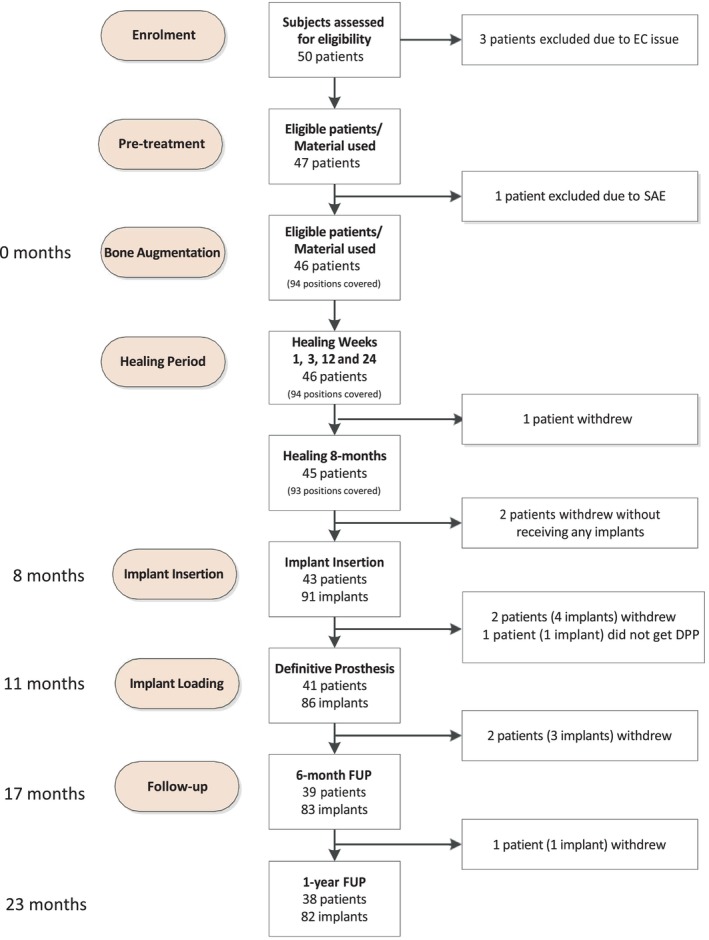
Treatment flow diagram according to STROBE. Timeline reflecting the study protocol.

A total of 50 subjects were initially enrolled across six participating centers; however, four subjects were excluded from the study prior to augmentation surgery. Figure 1 shows the study flowchart from enrollment to final analysis. The majority of patients were females, with a female: male ratio of nearly 2:1 ratio, and participants had a mean age of 50 years; therefore, a significant proportion of the study cohort were likely menopausal women with potentially associated osteoporotic issues. Bone quantity was rated as B or C for almost 80% of the study population. Detailed baseline characteristics of the study population are presented in Table 1.

**TABLE 1:** Baseline characteristics.

	*n* (%)	*n*‐assessed
Patients	46 (100%)	46
Sex		46
Female	30 (65.2%)	
Male	16 (34.8%)	
Age		46
Mean ± SD	50 ± 12.2	
Range	25–71	
Medical history		46
Periodontitis (controlled)	3 (6.5%)	
Diabetes type II (controlled)	1 (2.2%)	
Asthma	1 (2.2%)	
Allergies	6 (13%)	
Smoking habits		46
Never smoked	39 (84.7%)	
Past smoker	2 (4.3%)	
Current smoker ≤ 10 cigarettes/day	5 (10.9%)	
Bone quantity before augmentation		46
A	6 (13%)	
B	27 (58.7%)	
C	9 (19.6%)	
D	4 (8.7%)	
E	0 (0%)	
Bone quality before augmentation		46
1	15 (32.6%)	
2	24 (52.2%)	
3	7 (15.2%)	
4	0 (0%)	
GBR augmentation sites		119^b^
Augmented mandible positions		119^b^
First premolar	15 (12.6%)	
Second premolar	30 (25.2%)	
First molar	46 (38.6%)	
Second molar	27 (22.7%)	
Third molar	1 (0.8%)	
Implants, surgical approach, and prosthetics		91^a^
Implant distribution in the mandible^a^		91^a^
First premolar	13 (14.3%)	
Second premolar	21 (23.1%)	
First molar	39 (42.9%)	
Second molar	18 (19.8%)	
Implant ⌀ (mm)		91^a^
NP 3.75	21 (23.1%)	
RP 4.3	65 (71.4%)	
RP 5	5 (5.5%)	
Implant length (mm)		91^a^
8.5	39 (42.9%)	
10	40 (44%)	
11.5	11 (12.1%)	
13	1 (1.1%)	
Surgery type		91^a^
1‐stage surgery	42 (46.2%)	
2‐stage surgery	49 (53.8%)	
Bridge use		42
Yes	25 (59.5%)	
No	14 (33.3%)	
Not reported	3 (7.1%)	
Abutment type at DPP		86^a^
Esthetic abutment	18 (20.9%)	
Procera esthetic abutment	64 (74.4%)	
Temporary abutment	4 (4.7%)	

^a^ At implant level.

^b^ Guided bone regeneration (GBR) positions.

### 3.2 Primary Endpoint: Horizontal Bone Gain 8 Months After GBR

**FIGURE 2:**
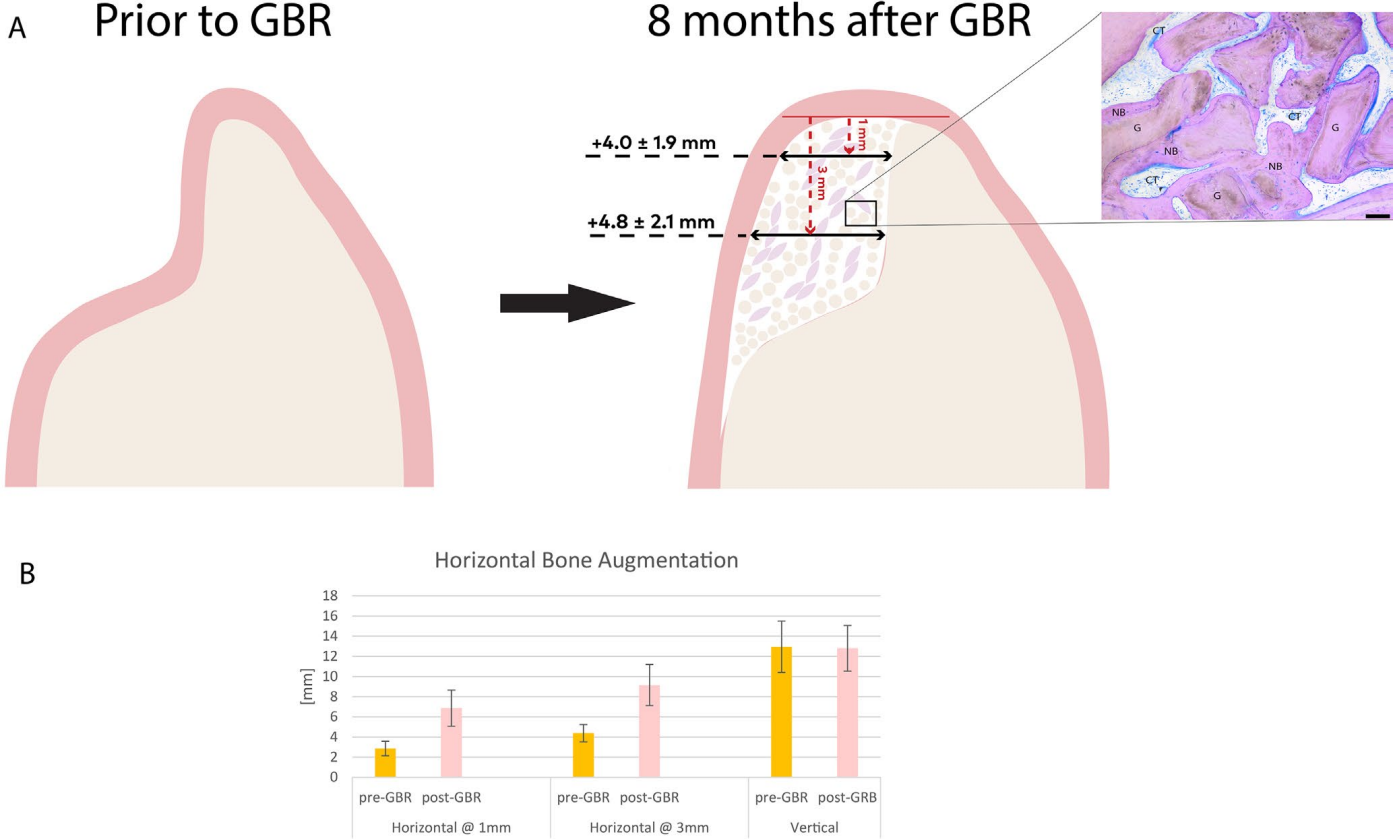
Illustration of the study's primary endpoint. Schematic drawing (A) depicting the ridge before (left) and after guided bone regeneration (GBR, right). The bone width gain was 4.0 ± 1.9 mm when measured 1 mm from the top of the crest and 4.8 ± 2.2 mm when measured 3 mm from the top of the crest. The inset shows the histological analysis of a trephine sample. CT, connective tissue; G, graft material; NB, new bone. The scale bar represents 100 μm. Bar graph (B) illustrates pre‐ and post‐augmentation measurements, error bars show standard deviations.

Bone augmentation outcomes were evaluated at the implant insertion visit (Figure 2). Of 45 patients evaluated for bone gain 8 months after GBR, 44 (97.8%) showed sufficient bone augmentation to allow for implant insertion, with nine patients (20%) recommended for additional simultaneous grafting. The single remaining case (2.2%) presented with insufficient RW to support implant placement. In this patient, a pre‐existing implant at FDI #47 had to be removed before performing augmentation procedures at site #45 due to severe bone loss. This patient was recommended for additional augmentation, and implant placement was postponed.

**FIGURE 3:**
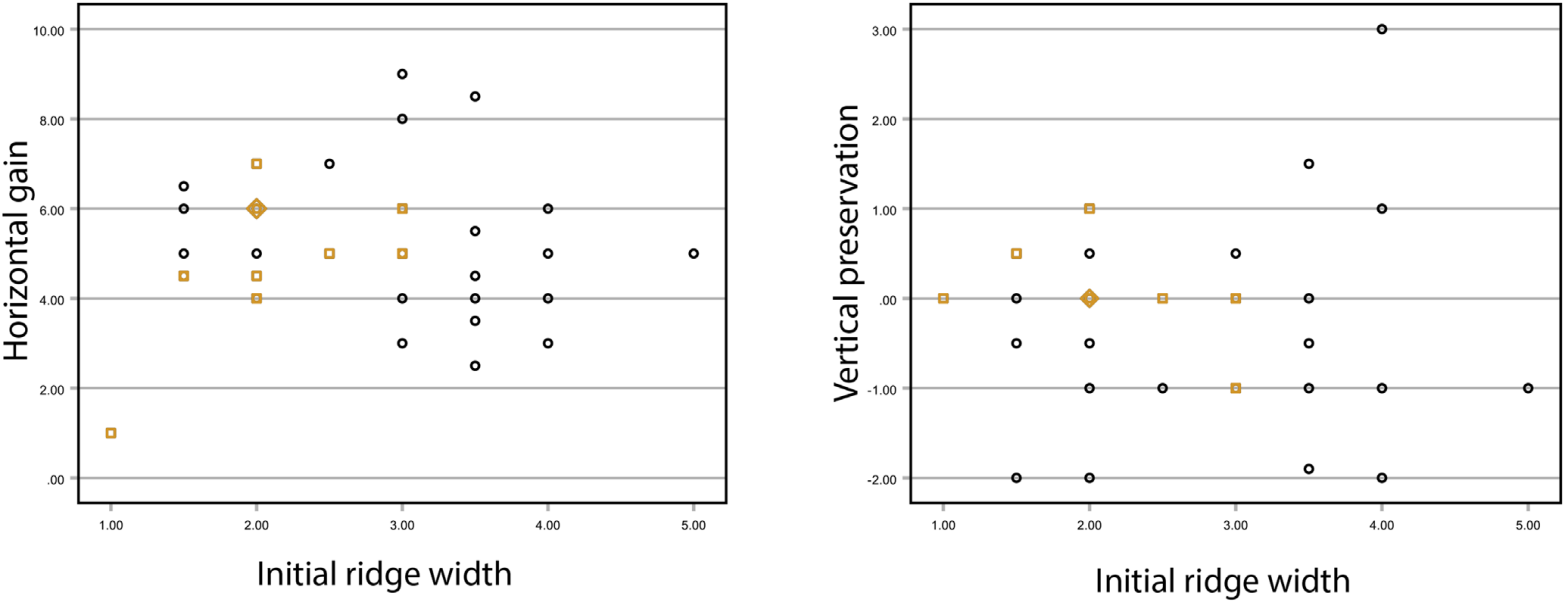
Initial ridge width vs horizontal bone gain or vertical preservation. The relationships between the pre‐treatment ridge width at 1 mm from the crest and the horizontal bone gain (left panel), and the vertical height preservation (right panel) are illustrated graphically. The sites that experienced dehiscence are marked as orange squares; the site with the implant failure is designated as an orange diamond, revealing that it was not an outlier in terms of defect size.

CBCT was performed at the time of the GBR procedure and 8 months later. After 8 months, a significant gain (*p* < 0.01) was observed. RW increased by 4.0 ± 1.5 mm (+140%) and 4.8 ± 1.7 mm (+109%) when assessed 1 mm and 3 mm from the top of the crest, respectively (Figure 2A). Although vertical gain was not a study objective, vertical height remained stable between the time of the GBR procedure and 8 months later, with a mean change of − 0.1 ± 2 mm (Figure 2B). The initial ridge width did not correlate with vertical preservation but showed a moderate correlation (*R* ^2^ = 0.706, *p* < 0.001) with horizontal bone gain (Figure 3).

### 3.3 Histological Evaluation

Prior to implant placement, a histological sample from the regenerated area was obtained by trephine. Because the trephine used was very thin in diameter, most of the obtained biopsies did not contain sufficient material for adequate analysis. Histological analysis from the seven readable samples revealed that they consisted of 37.3% residual graft, identified by the absence of cell nuclei; 33.6% vital bone formed around graft particulates; and 29% connective tissue (Figure 2, inset).

### 3.4 Soft Tissue Healing and Pain Assessment Following Bone Augmentation

**FIGURE 4:**
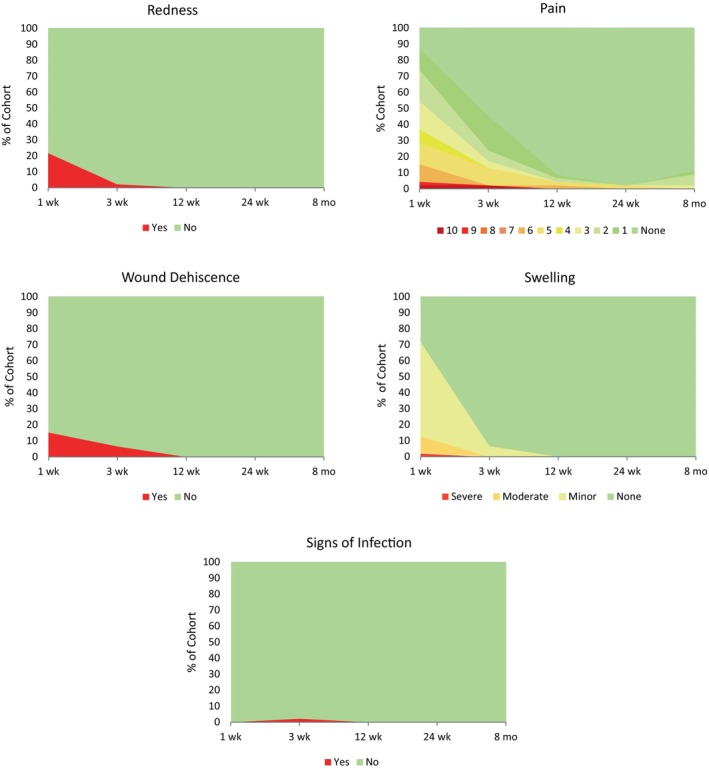
Soft tissue healing and pain perception during the healing period. Parameters were assessed from 1 week to 8 months post‐augmentation (X‐axis not to scale).

The analysis of soft tissue healing parameters, including swelling at the surgical site, flap sloughing, bleeding, local inflammation, infection, and pain, revealed few complications. Healing was judged by clinicians as uneventful (Figure 4); 87% of patients had minor (27) or no swelling (13) at 1‐week FUP, and 100% were deemed as having minor (3) or no swelling (43) by 3‐week FUP. No swelling was reported at 12 or 24 weeks. Mean perceived pain scores decreased from 3.1 ± 2.3 at 1‐week FUP to 0.3 ± 1.2 at 12‐week FUP and remained low thereafter (24‐week FUP: 0.1 ± 0.7; 8‐month FUP: 0.2 ± 0.7).

At 1‐week FUP, 10 patients (21.7%) presented with redness around the regenerated area. Pus was detected in one (2.2%) patient at 3 weeks, suggesting an infection. Wound dehiscence was observed in a total of 9 patients (19.6%): 7 (15.2%) and 3 (6.5%) patients at 1 and 3 weeks post‐GBR, respectively, with one patient displaying dehiscence at both visits. Four of these patients were indicated for additional bone grafting at the time of implant placement. Two patients did not receive additional DBBM material despite the initial indication and experienced implant failure: one due to the lack of osseointegration at 27 days post‐insertion and one due to radiolucency at 1 year. Of the three patients with dehiscence at 3 weeks, 2 (4.3%) presented with membrane exposure, one minor, and one size 10 × 3 mm. At 24 weeks, no complications were reported.

### 3.5 Implant Placement and Loading

**FIGURE 5:**
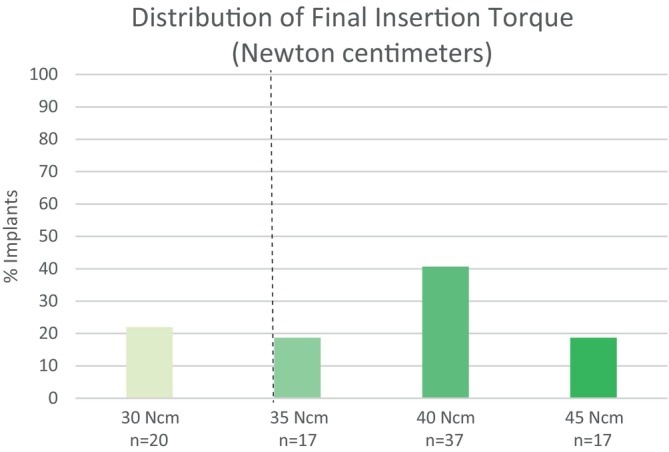
Distribution of final implant insertion torque values. Note that 78% of implants (right of the vertical dashed line) reached a sufficient torque to support immediate loading.

Of the 46 patients who underwent GBR, one withdrew prior to completing the evaluation for bone gain, and two withdrew prior to implant placement. Therefore, 43 patients received 91 implants in the augmented area, with 34 patients (79.1%) receiving two implants, 7 (16.3%) receiving three implants, and 2 receiving 1 implant (Table 1). The majority of implants (58.2%) were placed equicrestally, with the rest placed subcrestally. The mean final insertion torque was 37.8 ± 5.2 Ncm (range, 30–45 Ncm). Of 91 implants, 78% achieved a final insertion torque of ≥ 35 Ncm, a value considered sufficient for immediate loading (Figure 5). At the time of implant insertion, implant stability was also tested manually. Of 91 implants, 87 (95.6%) implants were reported as stable. The four remaining implants were initially determined as unstable by manual testing, however, because their insertion torque was between 35 and 45 Ncm, they were left in situ. Two of the four implants required concomitant bone grafting due to insufficient bone on the buccal site. All four implants subsequently osseointegrated and the patients successfully completed the study.

In approximately half of all cases (51.2% patients, 53.8% implants), implants underwent submerged healing; the remainder underwent 1‐stage surgery and received a healing abutment (*n* = 40) or an MUA (*n* = 2). Due to the retromolar location of the implants, neither immediate loading nor temporary solutions were necessary for esthetic reasons. At one study site, provisional prostheses were provided at the discretion of the treating clinician, with four patients receiving six prostheses placed 1.9 ± 1.1 months after implant insertion. DPP was performed in 41 patients for 86 implants 10.1 ± 3.9 months (range: 3.0–15.1 months) after implant insertion. Two patients with four implants withdrew from the study, and one patient did not receive a final prosthesis. The majority of patients received a Procera esthetic abutment (*n* = 64), and the remainder were fitted with either an angulated 17° MUA plus (*n* = 10), a straight MUA plus (*n* = 8), or a temporary abutment (*n* = 4). Because even favorably placed implants may not be 100% aligned with each other, especially parallel‐walled implants in posterior sites, angulated MUAs were used to provide more prosthetic flexibility in cases of multi‐unit restorations. Of 86 abutments placed, only 10 (8 in molars, and 2 in premolars) required angulated MUAs in 6 patients, and these only had 17° angulation. A bridge was used in 24 patients (59.5%), with the remaining patients either not using a bridge (33.3%) or not reporting it (7.1%). The 6‐month and 12‐month FUP visits occurred 6.1 ± 0.8 months (range, 3.9–8.8 months) and 12.2 ± 1.2 months (range, 9.3–15.9 months) after DPP, respectively.

### 3.6 Implant Survival and Success

The cumulative survival rate from implant insertion to 1‐year FUP was 98.9%; one implant failed to osseointegrate and was explanted prior to loading 27 days after implant insertion. The implant was replaced 49 days later but was no longer followed within this study. The cumulative implant success rate was 95.5% at 1‐year FUP; signs of radiolucency were observed for three implants in two patients at the last FUP. Kaplan–Meier curves illustrating survival and success are shown in Figure S1.

### 3.7 Radiographic Marginal Bone Levels and Bone Level Change Assessments

**FIGURE 6:**
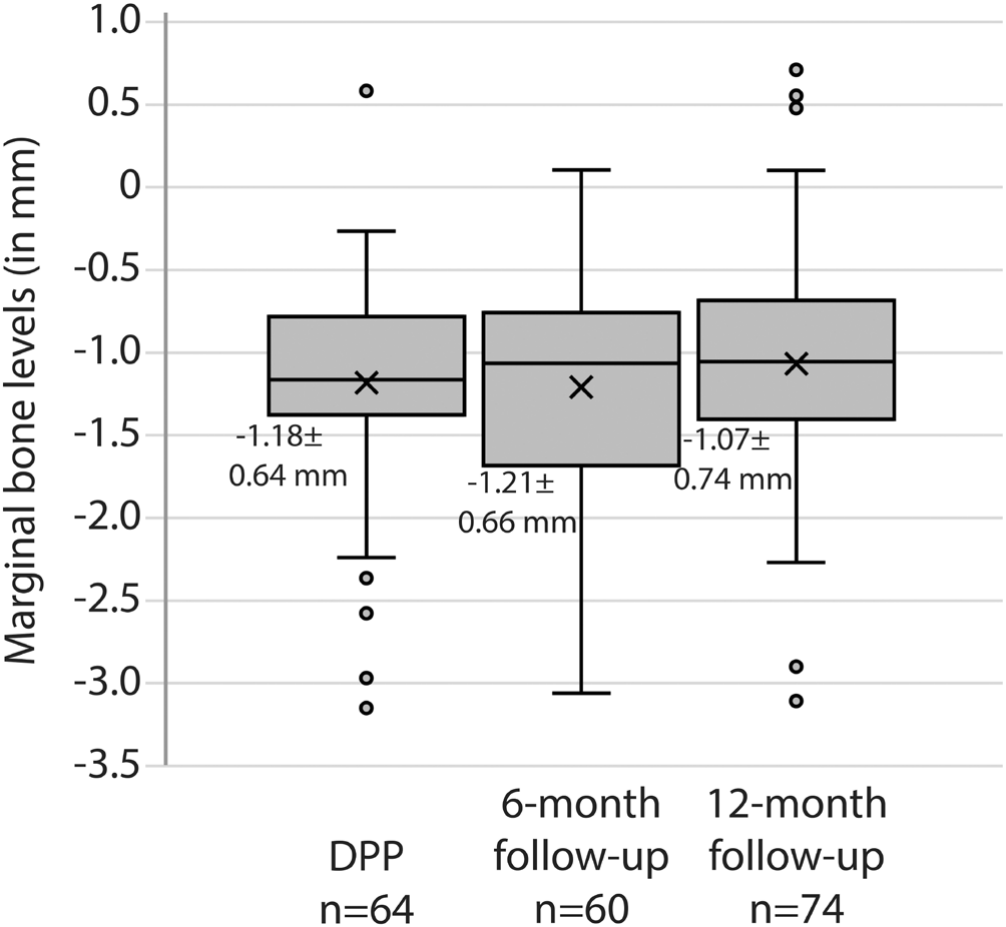
Marginal bone level from definitive prosthesis placement (DPP). The marginal bone level at DPP was −1.18 ± 0.64 mm and remained stable at 6‐month (−1.21 ± 0.66 mm) and 1‐year (−1.07 ± 0.74 mm) follow‐up.

Average MBL at DPP was − 1.18 ± 0.64 mm (*n* = 64 readable radiographs), and it remained stable over time with − 1.21 ± 0.66 mm (*n* = 60 readable radiographs) at 6 months and − 1.07 ± 0.74 mm (*n* = 74 readable radiographs) at 1 year (Figure 6). The mean MBL change (MBLC) was − 0.03 ± 0.55 mm (*n* = 56) from DPP to 6‐month FUP and − 0.03 ± 0.46 mm (*n* = 57) from DPP to 1‐year FUP. From 6 months to 1 year, the mean MBLC was − 0.01 ± 0.44 mm (*n* = 54). Crestal implant position at placement and 1‐ vs. 2‐stage surgery did not show a statistically significant influence on bone remodeling (Kruskal–Wallis test *p* = 0.381 and *p* = 0.734, respectively). One of the two subjects who experienced 3 mm marginal bone loss (1 site per patient) ultimately displayed radiolucency around the implant (FDI# 37).

### 3.8 Soft Tissue Assessment and Esthetics

**FIGURE 7:**
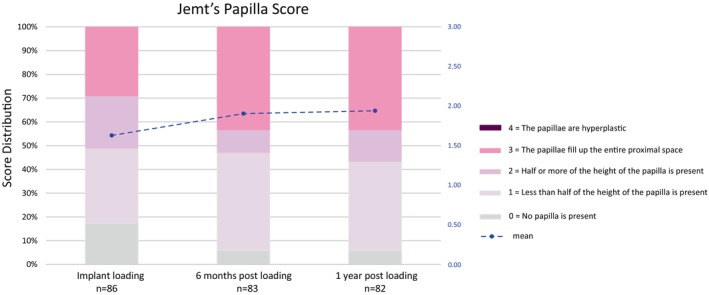
Jemt's Papilla score. Papillae at the mesial or distal sides of each implant position were evaluated. The score distribution is shown for the combined mesial plus distal values. The blue dashed line indicates the mean papilla score from definitive prosthesis placement to 1‐year follow‐up.

Soft tissue (mPl, mBI, Papilla Index, KM) and PES were assessed to determine health and appearance after GBR and implantation. Across all time points, more than 80% of implants showed no plaque accumulation or sulcus bleeding. No excessive bleeding was observed at any implants at any time point throughout the study. At DPP, 17.4% of implants had a Papilla Index score of 0, indicating no papilla, and only 29.1% of implants presented with a Papilla Index score of 3, which represents the optimal papilla contour. Most patients had Papilla Index scores of 1 (31.4%) or 2 (22.1%). By 6‐month FUP, the proportion of implants with no papilla decreased to 6%, whereas the proportion with optimal papilla rose to 43.4% and remained stable through 1‐year FUP (Figure 7). However, over 40% of implants did not achieve papilla heights of at least half, likely due to 60% of prosthetics being bridges, resulting in no papilla on an adjacent side due to the absence of a natural tooth. No implants presented with hyperplastic (score of 4) papilla at any point throughout the study.

The mean KM height decreased from 2.9 ± 1.5 mm at implant insertion to 1.8 ± 0.8 mm at DPP (Table 2) but remained stable thereafter. At implantation, five sites had no keratinized tissue and the proportions of implant sites with partially and fully keratinized tissue remained stable from DPP to 1‐year FUP. KM status was significantly associated (*p* < 0.001) with mBI scores, which were 0.00 for full KM, 0.11 for partial KM, and 0.63 for no KM.

**TABLE 2:** Keratinized mucosa (KM) score^a^ (% implants) and height in millimeters.

KM score (0–2)	Implant Insertion not assessed	DPP *n* = 86	6‐month FUP *n* = 83	1‐year FUP *n* = 82
0	N/A	18.6	16.9	15.9
1	N/A	31.4	27.7	31.7
2	N/A	50	55.4	52.4

KM height (mm)	Implant insertion *n* = 86	DPP *n* = 70	6‐month FUP *n* = 69	1‐year FUP *n* = 69
Mean	2.9	1.8	1.8	1.7
SD	1.5	0.8	0.8	0.7

^a^ 0 = no KM around implant, 1 = partial keratinization, 2 = full keratinization; FUP = follow‐up.

**FIGURE 8:**
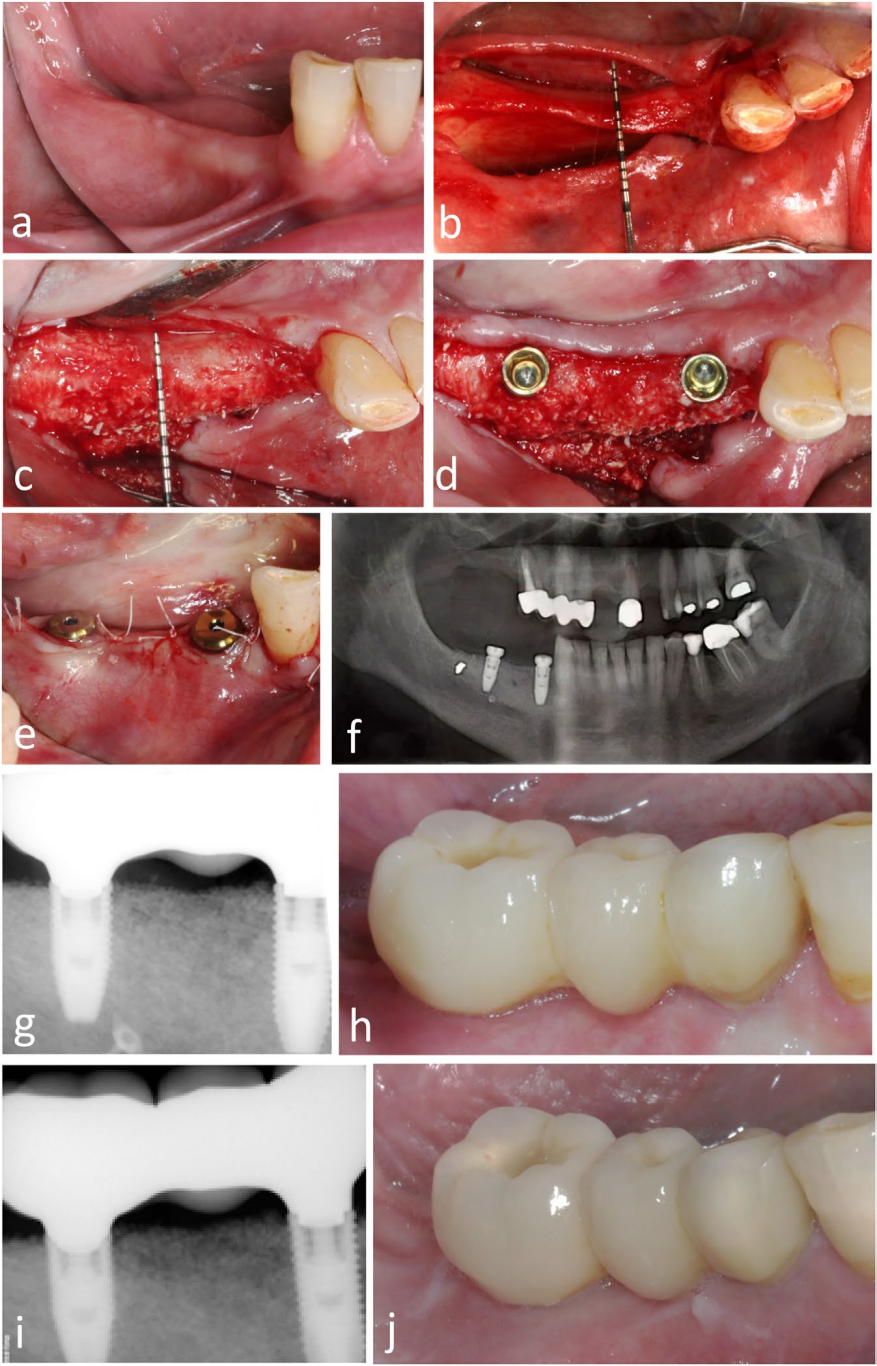
Sample clinical case. A 62‐year‐old woman presented with horizontal posterior mandible resorption (a). Ridge measurements were performed using a UNC 15 periodontal probe with millimeter marks placed in an occlusal view over the center of the augmentation site prior to (b) and 8 months after guided bone regeneration (GBR; c), followed by the placement of two parallel‐walled implants (d). A buccal clinical view (e) and a panoramic radiograph (f) were taken immediately after implant insertion and flap closure. Peri‐apical radiographs and clinical views at 6 months (g and h, respectively) and 1 year (i and j, respectively) after definitive prosthesis placement.

The mean PES at DPP was 7.6 ± 2.4 (range 2.0–13.0), it improved to 8.2 ± 2.1 (range 4.0–14.0) at 6 months and remained stable at 1 year (8.1 ± 2.0; range 4.0–14.0). A clinical case illustrates the treatment steps and FUP assessments (Figure 8).

### 3.9 Patient‐Reported Outcome Measures

OHIP‐14 questionnaire, which was administered throughout the study, revealed a notable improvement in patients' perceptions of oral health‐related quality of life after the augmentation procedure. The mean pre‐treatment score was 15.5 ± 11.5, which increased slightly during the healing phase, peaking at 19.4 ± 12.2 1‐week post‐GBR before falling to the pre‐GBR range after 12 weeks, indicating that GBR had a minimal impact on quality of life. At DPP, the score decreased to 9.1 ± 10.3 and continued to improve with the mean score of 5.4 ± 7.8 at 6 months and 3.9 ± 4.8 at 1 year. Patients lacking KM at the last FUP tended to report lower quality of life (*p* = 0.022).

Patients were highly satisfied with functional and esthetic outcomes. Mean functional satisfaction was 9.5 ± 1.0 at DPP, 9.6 ± 1.1 at 6 months, and 9.7 ± 0.8 at 1 year. Similarly, the mean esthetic satisfaction score was 9.7 ± 0.6 at DPP, 9.5 ± 1.0 at 6 months, and 9.6 ± 0.8 at 1 year.

### 3.10 Adverse Events

Minor adverse events were reported throughout the study. One patient lost an abutment, which was subsequently replaced, and another patient required three rounds of abutment replacement to identify an adequate abutment for optimal healing. The same patient presented with two missing pins, but the soft tissue healing was judged as uneventful and undisturbed. Soft tissues closed after the pins were lost without disrupting the procedure. In addition, 12 weeks post‐GBR, one patient experienced perforation by three pins on the lingual side, requiring pin removal and a single suture. In another case, a pin was visible but did not require any additional treatment.

### 3.11 Subanalysis

To assess the potential relationship between wound dehiscence and defect size, implant size and number per defect, and outcome measures, an additional descriptive analysis was performed. Overall, dehiscence was observed in 10 instances (21.7%) in 9 of 46 patients during the post‐GBR healing period. Most cases resolved before the 12‐week follow‐up without any intervention. Co‐occurring membrane exposure was reported for two cases (4.3%); one patient required a secondary stitch and the other required wound cleaning with CHX gel. The number of implants placed or the implant size did not correlate with the presence of wound dehiscence. However, the sites that experienced dehiscence tended to have a larger defect size, particularly when measured 3 mm from the crest. Specifically, the pre‐treatment RWs in these patients were 2.8 ± 0.7 mm and 3.9 ± 0.8 mm when measured 1 and 3 mm from the crest, respectively. GBR led to an increase in RW by 3.2 ± 1.0 mm at 1 mm and 4.1 ± 1.7 mm at 3 mm at these sites, all of them (*n* = 9) were considered sufficient for implant placement and received 17 implants with a mean final insertion torque of 40.9 Ncm. Of the 17 implants, 1 failed, and 2 were deemed unsuccessful. Figure 3 provides detailed information on horizontal bone gain and vertical height maintenance for sites with dehiscence.

Soft tissue outcomes differed to some degree for the group with wound dehiscence. At DPP, 18.8% of implants had a Papilla Index score of 0, indicating no papilla, and 18.8% of implants presented with a Papilla Index score of 3, which represents the optimal papilla contour. Most patients had Papilla Index scores of 1 (37.5%) or 2 (25.0%). Although implants with no papilla decreased to 0% at 6‐month FUP, the proportion with optimal papillae did not increase, and some that previously had a score of 2 regressed to a score of 1, which became the predominant fraction (62.5%). These scores remained stable through 1‐year FUP. As the study progressed, the mPI among the dehiscence group improved, with the proportion showing no accumulation increasing from 81.3% at DPP to 93.8% at 6‐month FUP and 100% at 1‐year FUP. Similarly, the mBI improved, with the proportion of implants displaying no bleeding in the surrounding tissue increasing from 87.1% at DPP to 100% at 1‐year FUP (with a slight dip to 81.3% at 6‐month FUP). The mean KM height decreased from 3.3 ± 2.0 mm at implant insertion to 1.6 ± 1.1 mm at DPP before improving to 1.9 ± 0.7 mm at 6‐month FUP and remaining stable thereafter. At DPP, 1 site had no keratinized tissue, 10 had partially‐ and 5 fully keratinized mucosa. At 6‐month FUP, twice as many implant sites had full KM (10) than had partial KM, which remained stable at 1‐year FUP. The mean PES at DPP was 7.5 ± 2.7 (range 3.0–13.0), which improved to 7.9 ± 2.1 (range 4.0–13.0) at 6‐month FUP and remained stable at 1‐year FUB (7.8 ± 2.0; range 4.0–13.0).

**FIGURE 9:**
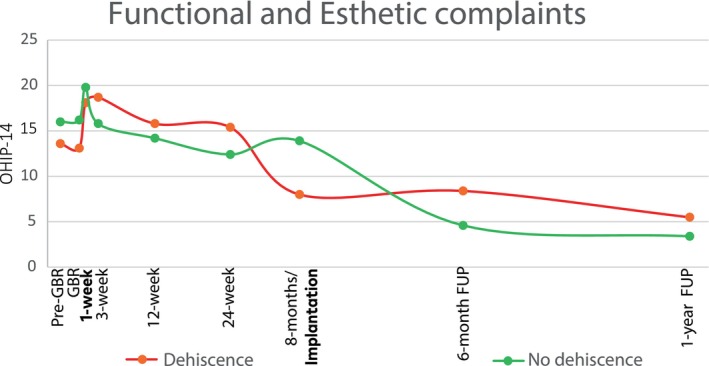
Quality of life (QoL) in patients with dehiscence. An increase in OHIP‐14 scores reflects the perceived worsening of QoL, which is expected directly after invasive procedures. However, complaints remained higher among those who experienced dehiscence than among those who did not experience dehiscence.

Although patient perceptions of oral health‐related quality of life improved overall throughout the study, temporary worsening was recorded at 1‐week post‐GBR and implant placement (indicated by elevated OHIP‐14 scores). In general, patients rebounded to the pre‐treatment state and beyond; however, complaints remained higher among the dehiscence subgroup than among the general population throughout the 24‐week healing period (Figure 9).

## 4 Discussion

This study demonstrated that the use of DBBM combined with non‐crosslinked resorbable collagen membrane in the GBR procedure was accompanied by uneventful healing leading to a bone defect repair and greater than 100% width gain, with moderate correlation between initial defect size and bone gain. In the few readable samples, histological assessment revealed bone graft material ossification. In most cases, regenerated bone allowed for successful implant placement, with a high survival rate (98.9%), stable MBL from DPP to 1‐year FUP, a healthy soft tissue response, and acceptable esthetics, as assessed by the Papilla Index, mBI, mPI, KM, and PES scores.

The quantity of mean bone gain after GBR was 4 mm when the RW was measured 1 mm from the top of the crest and 4.8 mm when measured 3 mm from the top of the crest, which are slightly higher values than the overall average of 2.27 mm reported in a systematic review encompassing 20 studies (Aleksic et al. 2018; Wessing, Lettner, and Zechner 2018). However, this review included investigations using various grafting materials, including anorganic bovine bone material, autologous bone chips, demineralized bone matrix, freeze‐dried bone allograft, and combinations thereof, combined with either non‐cross‐linked or crosslinked collagen membranes. Because graft particle size can influence vascularization and bone healing rates (Leiblein et al. 2020; Sanz et al. 2019), a suitable membrane aids in long‐term volume stability and enduring resistance against degradation, even without chemical crosslinking (Shin et al. 2014). The technique itself can also have a profound impact. For example, clinicians may pack grafts too tightly when attempting to stabilize the regenerated ridge defect, but the over‐compression of the interconnected macropores in the graft material may prevent the invasion of cells and capillaries, hindering the vascularization and liquid uptake required for nutrient flow (Bozkurt et al. 2014; Fernandez de Grado et al. 2018; Lee et al. 2010; Zhang et al. 2018). Conversely, since adequate compression is necessary to provide stability and prevent granule movement, when insufficient, it could compromise the structural integrity of the graft. In addition, membrane fixation with titanium pins is essential for ensuring that the graft remains in place when using a bone matrix, particularly when repairing ridge defects (Mir‐Mari et al. 2016; Reddy et al. 2019; Redemagni, Mascetti, and Garlini 2019; Wessing, Emmerich, and Bozkurt 2016; Wessing et al. 2017). In the current study, the clinical investigation plan instructed surgeons to use titanium pins to fix the resorbable collagen membrane for greater stability.

In GBR, the membrane serves as a natural barrier that protects the space from the undesirable migration of soft tissue while allowing the ingrowth of osteogenic cells, creating an environment that is favorable for bone formation (Jager et al. 2007; Jager et al. 2006; Rodriguez et al. 2018; Villa and Rangert 2007). Therefore, a membrane that allows the movement of select cells and nutrients is preferable to a complete barrier (Omar et al. 2018; Wang, Lin, and Kang 2019).

A very low rate of complications was observed in the current study, which aligns with previously published analyses (Aleksic et al. 2018; Bozkurt et al. 2014; Lim et al. 2018; Omar et al. 2018; Sanz‐Sanchez et al. 2022; Sanz Sánchez et al. 2018; Wessing, Emmerich, and Bozkurt 2016; Wessing et al. 2017). A systematic review assessing complications associated with GBR in five studies reported a site‐level weighted mean incidence rate of 9.9% for minor wound dehiscence, whereas minor infections occurred at a rate of 1.5%. A patient‐level analysis found that minor complications occurred at a weighted mean incidence rate of 16.1%, compared with 1.6% for major complications (Tay et al. 2020). Except for a few minor AEs, most patients treated with GBR experience uneventful wound healing, largely free of inflammation, swelling, redness, or infection (Bozkurt et al. 2014), in line with the uneventful healing observed in this study.

The overall dehiscence rate in the current study was 19.6%, which is comparable to the range of 4%–26% reported in a prior study (Wessing et al. 2017). All dehiscence cases in the current study resolved quickly with no further incidents after the third week of healing. The use of a resorbable, non‐crosslinked collagen membrane may have contributed to the low dehiscence rate and the rapid resolution of dehiscence cases. This membrane is characterized by minor surface expansion, which may reduce the strain placed on the primary wound closure. The use of a less expandable membrane, such as the membrane used in the current study, could reduce the risks of prolonged dehiscence and potential procedural failure in cases of severe defects (Arrighi et al. 2014; Wessing, Emmerich, and Bozkurt 2016). In addition, the resorbable collagen membrane used in the current study has excellent suture retention and high resistance to tearing, facilitating the fixation of the membrane using either sutures or pins (Arrighi et al. 2014; Bozkurt et al. 2014; Gasser et al. 2016; Wessing, Emmerich, and Bozkurt 2016). These features were reflected in the high success rate observed in this study, with only a few reports of pin loss or perforation, none of which had clinical consequences.

The post hoc descriptive analysis performed for the nine cases that experienced wound dehiscence revealed that this group had lower ridge width after the GBR procedure. However, these cases also tended to have larger initial bone defects. The dehiscence group also showed lower Papilla Index scores and a longer (albeit temporary) decrease in oral health‐related quality of life during the healing phase than the non‐dehiscence group. Due to the low overall number of dehiscence cases, our study did not have sufficient statistical power to assess the impact of dehiscence on clinical outcomes. Nevertheless, this study suggests that dehiscence may have a negative impact, particularly on patient satisfaction.

Wound dehiscence that results in membrane exposure may have a detrimental effect on bone gain (Cadenas‐Vacas et al. 2021; De Bruyckere et al. 2018; Machtei 2001; Redemagni, Mascetti, and Garlini 2019; Wessing, Emmerich, and Bozkurt 2016; Wessing et al. 2017). Previous studies suggest that exposed membranes have deleterious impacts on GBR, where augmentation procedures without exposure achieve a 74% increase in horizontal bone gain compared with those that experience exposure. In cases of dehiscence, sites without exposure displayed a 27% increase in defect reduction compared with sites with exposure (Aleksic et al. 2018; Bouguezzi et al. 2020; Garcia et al. 2018; Wessing, Lettner, and Zechner 2018). In the current study, two patients (4.3%) experienced membrane exposure, one of whom withdrew due to an unrelated adverse event (a digestive illness). This result is in agreement with a systematic review reporting that non‐crosslinked membranes experience a 30% lower rate of membrane exposure relative to crosslinked membranes and are less likely to induce an adverse reaction during resorption. The number of membrane exposure cases observed in the current study was too low to permit any conclusions regarding the effects of membrane exposure on bone gain or other clinical outcomes.

The GBR in this study facilitated sufficient bone regeneration to support successful implant placement. Most implants in the study achieved a high final insertion torque, reflecting the good quality of regenerated bone. At 1 year post‐loading, the implant survival rate was 98.9%, which is comparable to the rates of 100% reported by both Wessing et al. (2017) and Urban et al. (2019). The one failure to osseointegrate occurred in a patient with prior heavy smoking habits who failed to maintain good oral hygiene and presented with dehiscence at weeks 1 and 3. This patient eventually underwent additional bone grafting but received only autologous bone chips and a collagen membrane without DBBM inclusion. In addition to this failed implant, three implants in two patients were classified as unsuccessful due to radiolucency at 1 year. The patient with radiolucency around two implants presented with dehiscence at Week 1, and although the subject was initially indicated for concomitant bone augmentation at implant insertion, during the implant placement surgery, the implant was found to be fully surrounded by bone, and the decision was made to proceed without additional bone grafting. The other patient with radiolucency around one implant had a history of periodontitis, implying that habits and pre‐existing conditions may have affected both implant survival and success.

The MBLC remained stable from DPP to both 6‐month and 1‐year FUP, and the overall average was comparable to the mean calculated in a meta‐analysis of studies with the same FUP period (Kumar et al. 2021). The observed decrease in KM height after implant insertion was analogous to a prior report evaluating the tissues around teeth compared with those around implants (Chang et al. 1999). A recent meta‐analysis of soft tissue status following GBR suggested that initial considerations of KM quantity, flap thickness, and tension could determine the occurrence of complications during the recovery. Thus, overall soft tissue management may play a pivotal role in the ultimate outcome (Lim et al. 2018). In addition to functionality, healing and esthetics increasingly drive treatment objectives due to a rising emphasis on patient satisfaction, and PROMs illustrate the process of care to more than just the final result (McGrath, Lam, and Lang 2012). In the current study, the OHIP‐14 questionnaire detected a brief decrease in quality of life during the healing process, which returned to pre‐treatment levels by 12 weeks after most complications resolved, and it reflected high satisfaction levels immediately after DPP. Although a clinical study comparing early and delayed implant placement suggested that delayed placement may carry an initial risk of missing papillae, this deficiency appears to resolve within 18 months (Schropp et al. 2005). A review reported that 60% of implant sites have a Papilla Index score of 2, which is similar to the present study, in which almost 60% of cases attained scores of 2 (≥ half of papilla) or 3 (optimal) by 12 months (Chen and Buser 2009).

The main limitations of this study include the relatively short follow‐up period, an insufficient sample size to determine the effects of wound dehiscence or defect type on clinical outcomes, the use of an uncommon definition of implant success, and few readable histological samples, which may not represent a comprehensive analysis of GBR results at a cellular level. The current study conducted follow‐up examinations for only 1‐year post‐DPP. Although the first year is usually the most critical period in terms of implant survival and success, an extended monitoring period would be essential to verify the stability and capacity of the bone graft to support long‐term outcomes. Regarding the success criteria, this study applied the definition of van Steenberghe, which may now be outdated but was originally selected for the purpose of providing a comparison with prior studies. Finally, although the current study explored the potential impact of wound dehiscence, a larger number of such cases should be investigated to provide sufficient statistical power.

## 5 Conclusion

This prospective study demonstrated that horizontal bone defects augmented with DBBM and guided by a non‐crosslinked resorbable collagen membrane led to efficient bone regeneration, allowing successful implant placement while maintaining healthy and stable responses by peri‐implant hard and soft tissues. Within the limitations of the single‐arm cohort study, this grafting modality offered partially edentulous patients a predictable treatment course with uneventful healing and, notably, improved their quality of life, leading to high satisfaction with both function and esthetics.

## Author Contributions

**Jonas Lorenz:** investigation, writing – review and editing, formal analysis. **Shahram Ghanaati:** investigation, writing – review and editing. **Zoran Aleksic:** investigation, writing – review and editing. **Iva Milinkovic:** investigation, writing – review and editing. **Zoran Lazic:** investigation, writing – review and editing. **Marko Magić:** investigation, writing – review and editing. **Bastian Wessing:** investigation, writing – review and editing. **Ramona Schleich Grotenclos:** investigation, writing – review and editing. **Mauro Merli:** investigation, writing – review and editing. **Giorgia Mariotti:** investigation, writing – review and editing. **Eriberto Bressan:** investigation, writing – review and editing. **Luca De Stavola:** investigation, writing – review and editing. **Robert Sader:** investigation, writing – review and editing, formal analysis.

## Conflicts of Interest

Bastian Wessing and Luca De Stavola formerly served as Key Experts for NBS. Other authors declare no conflicts of interest.

## Supporting information


**Figure S1** Kaplan–Meier curves for implant survival (top) and implant success according to the van Steenberghe criteria (bottom).

## Data Availability

The data that support the findings of this study are available from the study sponsor upon reasonable request.
